# Action of the Academy of Medicine

**Published:** 1875-04

**Authors:** 


### Editorial and Miscellaneous.

*Action of the Academy of Medicine.*

As a matter of information to our readers, we are authorized to state that the charges, specifications, and proof upon which Dr. John G. Westmoreland was tried and expelled from the Middle Georgia Medical Association, were forwarded to the Atlanta Academy of Medicine for action, and upon which charges, etc., Dr. J. G. Westmoreland was expelled, by a unanimous vote, from the Atlanta Academy of Medicine. We are further authorized to state that the matter will be carried to the State Medical Association, where, it is understood, Dr. Westmoreland will defend his action upon which the charges, etc., were predicated.

